# Bee xinmovirus 1 near-complete genome sequenced from honey bees and mining bees in Israel

**DOI:** 10.1128/mra.00960-25

**Published:** 2026-02-05

**Authors:** Boone H. Jones, Katie F. Daughenbaugh, Charles C. Carey, Idan Kahnonitch, Tal Erez, Na'ama Arkin, Asaf Sadeh, Nor Chejanovsky, Yael Mandelik, Michelle L. Flenniken

**Affiliations:** 1 Department of Microbiology and Cell Biology, Montana State University 33052, Bozeman, Montana, USA; 2 Pollinator Health Center, Montana State University 33052, Bozeman, Montana, USA; 3 Department of Entomology, Institute of Environmental Sciences, Faculty of Agriculture, The Hebrew University of Jerusalem 26742, Rehovot, Israel; 4 Department of Agroecology and Natural Resources, Newe Ya'ar Research Center, Agricultural Research Organization Volcani Institute 42718, Ramat Yishay, Israel; 5 Department of Entomology and Nematology, Institute of Plant Protection, Agricultural Research Organization - Volcani Institute 42718, Rishon Letzion, Israel; 6 The Mina & Everard Goodman Faculty of Life Sciences, Bar Ilan University 108397 , Ramat Gan, Israel; 7 Department of Plant Sciences and Plant Pathology, Montana State University 33052, Bozeman, Montana, USA; Katholieke Universiteit Leuven, Leuven, Belgium

**Keywords:** RNA virus, honey bee, mining bee, xinmovirus, *Xinmoviridae*

## Abstract

Sequence data indicate that a new virus, bee xinmovirus 1, is present in both honey bee (*Apis mellifera*) and mining bee (*Andrena* spp.) samples from Israel. The near-complete genome is approximately 13,130 nucleotides long and most similar to viruses of the family *Xinmoviridae* (S. Sharpe and S. Paraskevopoulou, J Gen Virol 104:001906, 2023, https://doi.org/10.1099/jgv.0.001906).

## ANNOUNCEMENT

**Fig 1:**
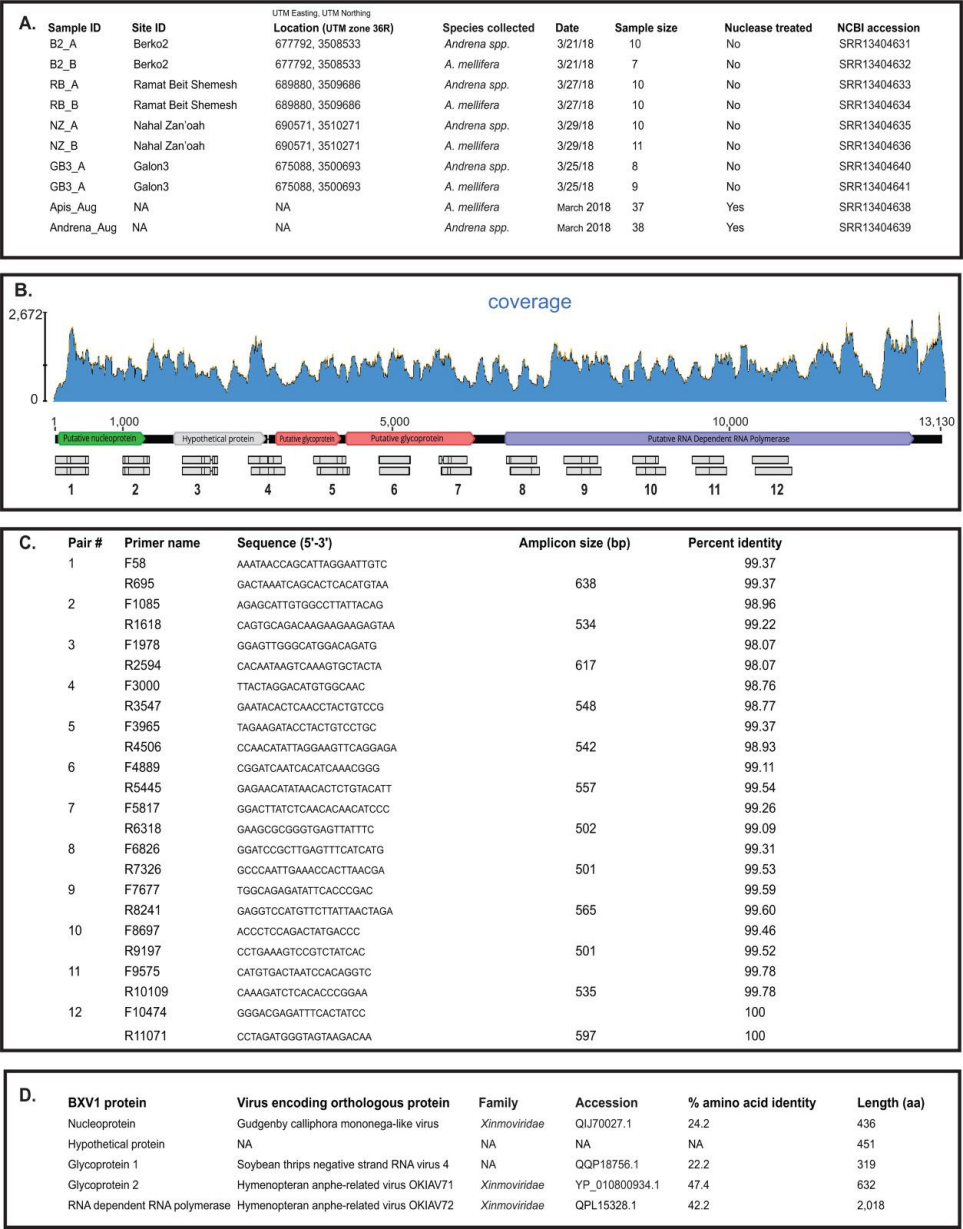
Bee xinmovirus 1 near-complete genome obtained from bees sampled in Israel. (**A**) *Apis mellifera* and *Andrena* spp. samples were obtained from four sites in Central Israel as described by reference 11. The table includes additional information on the samples and sequences, which were utilized for the bioinformatic analyses reported herein. (**B**) The nearly complete bee xinmovirus 1 (BXV1) genome (PV899697) coverage map generated from read alignments of the honey bee sequencing library SRR13404634 shows high viral abundance, with maximum and mean coverage depths of ~2,672× and ~1,075× , respectively (blue shaded region). The BXV1 near-complete genome has five predicted open reading frames that encode the putative nucleoprotein (green), hypothetical protein (gray), two glycoproteins (pink), and RNA-dependent RNA polymerase (purple). Approximately 50% of the BXV1 near-complete genome was Sanger-sequenced using cDNA from a subset of the individual BXV1-positive honey bee samples (*n* = 3) (gray rectangles below genome indicate identical sequence, and mismatches are indicated with black lines) utilizing the primer pairs listed in (**C**). Primer names denote forward or reverse, followed by starting position on the BXV1 genome and amplicon size. Percent nucleotide identity of Sanger sequence relative to assembled BXV1 genome is given, ranging from 98.07% to 100%. (**D**) Putative BXV1 genome encoded proteins were identified by homology to known viral proteins (BLASTx).

Honey bees (*Apis mellifera*) are commonly infected with viruses (1–3). Recent sequencing efforts have expanded the bee virome and indicate that many viruses that were first characterized in honey bees have a broader host range (3–10). Herein, we re-examined sequence data obtained from co-foraging honey bee and mining bee (*Andrena* spp.) samples collected from four sites in central Israel (Bioproject PRJNA687318) (11, 12). In brief, bees were homogenized in an aqueous buffer. One pooled sample for each species (i.e., honey bee [*n* = 37], mining bee [*n* = 38]) was treated with nucleases prior to RNA extraction to degrade host nucleic acids, generating two species-specific virus-enriched samples (Fig. 1A) (11, 13). Additionally, four site-specific pooled samples were generated for each species (*n* = 7–11) (Fig. 1A). RNA was extracted using TRIzol reagent. Libraries were prepared using the Illumina TruSeq Stranded RNA Sample Prep Kit and sequenced on an Illumina HiSeq 4000. Approximately 45–55 million (2 × 100 bp paired end) reads were generated for each of the eight samples representing each site by species and 12–14 million reads for each of the two species-specific virus-enriched libraries (11). Daughenbaugh et al. reported sequence variants of bee-infecting viruses and identified and characterized Andrena-associated bee virus 1 (11). To further explore honey bee and mining bee viromes, analyses of the two virus-enriched libraries using bioinformatic tools were performed using default parameters. Specifically, reads were trimmed using BBduk (v.39.26) (13). Reads that aligned to the honey bee genome (Amel_HAv3.1 NCBI) and the Holobee Index, which include genomic sequences from 55 bacteria, five fungi, six mites, one protozoan, and 86 viruses, using HISAT2 (v.2.2.1) were removed from analyses to facilitate virus discovery (11, 14–18). Reads from the two virus-enriched libraries were assembled using TRINITY (v.2.15.2) and putative viral contigs were identified by DIAMOND BLASTx alignment to the NCBI Virus RefSeq protein database (r.299) (19, 20). The most prevalent contig in the honey bee and mining bee virus-enriched libraries was further characterized and named bee xinmovirus 1 (BXV1) (BioProject PRJNA687318) (11). The consensus sequences for BXV1 from *A. mellifera* (PV899697) and *Andrena* spp. (PX227287) were determined by aligning reads from two libraries with abundant BXV1 (i.e., SRR13404634—142,193 reads, SRR13404635—654,763 reads) (Fig. 1B). The BXV1 near-complete genome is at least 13,130 nucleotides long with a GC content of 32.41% (Fig. 1B). To verify the assembly, approximately 50% of the genome was Sanger-sequenced using cDNA from a subset of individual BXV1-positive honey bee samples (Fig. 1C). The BXV1 sequences from *Andrena* spp. and *A. mellifera* samples were 99.4% identical, indicating that the virus was shared among co-foraging bee species. Five open reading frames over 300 amino acids long were predicted to encode the RNA-dependent RNA polymerase, two glycoproteins, a hypothetical protein, and a nucleoprotein (Fig. 1D). Based on homology to viruses of the family *Xinmoviridae*, the near-complete genome is putatively negative sense single-stranded RNA (21). Future studies will include further characterization of the virion, host range, and potential impacts of this virus on bee hosts.

## Data Availability

As part of a previous study, species-specific sequencing data were deposited under BioProject ID PRJNA687318 (11). The near-complete BXV1 genome sequences from *A. mellifera* and *Andrena* spp. were deposited in GenBank with accession numbers PV899697 and PX227287, respectively. Sanger sequencing reads of the BXV1 genome were deposited in the GenBank Short Read Archive, Bioproject PRJNA1310646.
